# Modified Aminoglycosides Bind Nucleic Acids in High-Molecular-Weight Complexes

**DOI:** 10.3390/antibiotics9020093

**Published:** 2020-02-21

**Authors:** Lanqing Ying, Hongkun Zhu, Marina Y. Fosso, Sylvie Garneau-Tsodikova, Kurt Fredrick

**Affiliations:** 1 Department of Microbiology and Center for RNA Biology, The Ohio State University, Columbus, OH 43210-1292, USA; lqying567@gmail.com (L.Y.); hongkun.zhu@gmail.com (H.Z.); 2 Department of Pharmaceutical Sciences in the College of Pharmacy, University of Kentucky, Lexington, KY 40536-0596, USA; marina.fosso@uky.edu (M.Y.F.); sylviegtsodikova@uky.edu (S.G.-T.)

**Keywords:** tobramycin, kanamycin, RNA, DNA, reverse transcriptase

## Abstract

Aminoglycosides represent a large group of antibiotics well known for their ability to target the bacterial ribosome. In studying 6”-substituted variants of the aminoglycoside tobramycin, we serendipitously found that compounds with C_12_ or C_14_ linear alkyl substituents potently inhibit reverse transcription in vitro. Initial observations suggested specific inhibition of reverse transcriptase. However, further analysis showed that these and related compounds bind nucleic acids with high affinity, forming high-molecular weight complexes. Stable complex formation is observed with DNA or RNA in single- or double-stranded form. Given the amphiphilic nature of these aminoglycoside derivatives, they likely form micelles and/or vesicles with surface-bound nucleic acids. Hence, these compounds may be useful tools to localize nucleic acids to surfaces or deliver nucleic acids to cells or organelles.

## 1. Introduction

Aminoglycosides represent a group of structurally diverse amino-modified polysaccharides that are well known for their broad-spectrum activity against bacteria. Most aminoglycosides are structurally similar and share a central 2-deoxystreptamine ring. These compounds (henceforth abbreviated “AGs”) bind helix h44 of 16S ribosomal RNA (rRNA) and inhibit protein synthesis [1,2], leading to bacterial cell death. AGs also interact with a number of other RNA targets. These include secondary rRNA sites [3], natural riboswitches [4], group I introns [5], and several retroviral RNA elements [6,7,8,9]. Binding stems from the ability of these positively-charged amino-rich AGs to form electrostatic interactions with the negatively-charged phosphate groups of the RNA [10], and to a lesser extent from hydrogen bonding involving the amino and hydroxyl groups of AGs [11]. The conformation of AGs along with the size of the interior loop of the target RNAs also influence the binding affinity [12,13]. 

In addition to RNA, AGs have been shown to target certain proteins. AG-arginine conjugates (semi-synthetic AGs in which l-arginine moieties are attached to parent AGs) have been shown to prevent HIV-1 Tat protein from binding the transactivation responsive element [14] or interacting with gp41 and gp120 proteins [15,16]. These compounds have also been shown to inhibit early stages of the virus life cycle [17,18].

## 2. Results

### 2.1. Potent Inhibition of Reverse Transcriptase by Certain Aminoglycoside Variants

**Figure 1:**
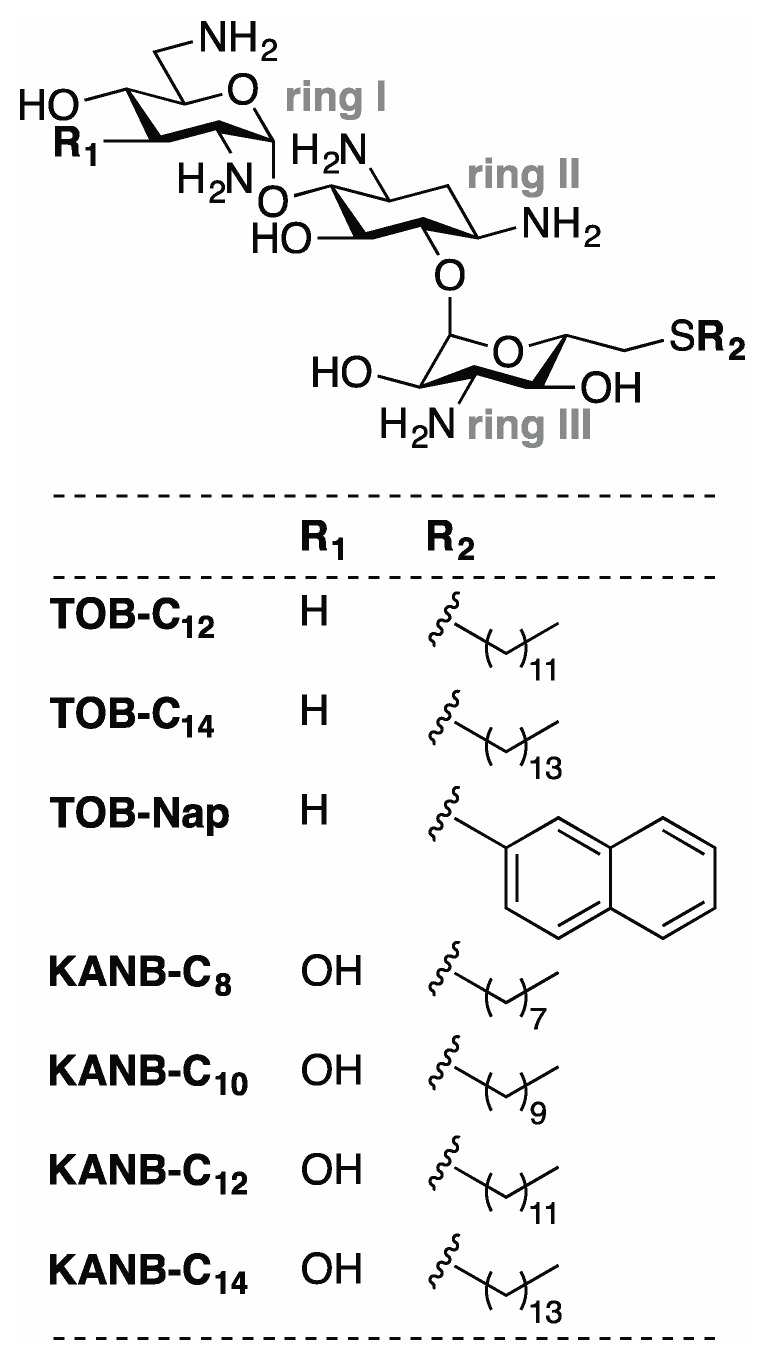
Compounds analyzed in this study. Related aminoglycosides TOB and KANB were modified with various 6”-thioether substituents to generate the compounds shown. Derivatives of TOB (TOB-C_12_, TOB-C_14_, TOB-Nap) and KANB (KANB-C_8_, KANB-C_10_, KANB-C_12_, KANB-C_14_) differ chemically at two positions (R_1_ and R_2_) as indicated.

**Figure 2:**
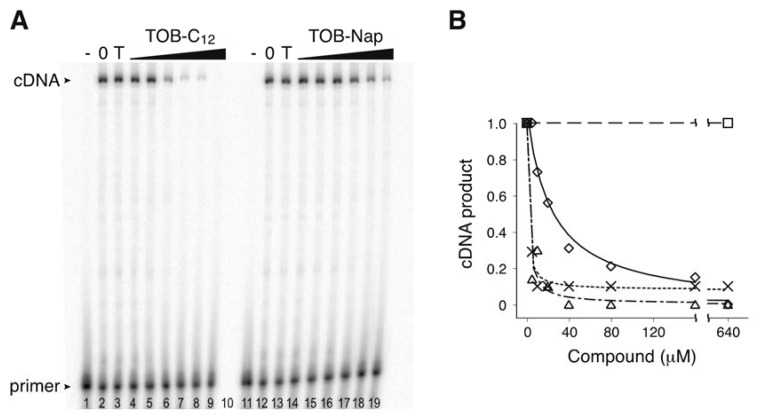
TOB variants inhibit AMV RT. (**A**) An experiment measuring primer extension in the presence and absence of TOB derivatives. Radiolabeled oligonucleotide primer was annealed to an RNA template and added to AMV RT and dNTP substrates in the absence (“0” lanes 2 and 12) or presence of TOB (“T” lanes 3 and 13; 1000 µM) or its derivatives (TOB-C_12_, TOB-Nap; increasing concentrations as indicated). A control reaction lacking RT (“-” lanes 1 and 11) was included. Following incubation for 10 min at 37 °C, reaction products were resolved by PAGE. (**B**) The relative amount of full-length cDNA product was plotted as a function of compound concentration (☐, TOB; △, TOB-C_12_; ✕, TOB-C_14_; ◇, TOB-Nap), and the data were fit to a modified dose-response equation to obtain IC_50_ values (TOB-C_12_, 1.6 µM; TOB-C_14_, 1.1 µM; TOB-Nap, 23 µM).

Using the toeprinting technique [19] to study the effects of various 6"-thioether tobramycin (TOB) derivatives on ribosomal translocation [20], we were surprised to observe no primer extension products in some reactions. This suggested inhibition of avian myeloblastosis virus (AMV) reverse transcriptase (RT) by some of these compounds (Figure 1), which was confirmed in subsequent experiments (Figure 2). Those TOB derivatives with clear inhibitory activity have naphthyl or linear alkyl (C_10_–C_22_) moieties, and the most potent inhibitors (termed TOB-C_12_ and TOB-C_14_; IC_50_ values ~1 µM) bear C_12_ and C_14_ linear alkyl groups.

Reverse transcriptases share structural similarity to the single-subunit polymerases of bacteriophage T7 [21,22,23,24]. To assess specificity, we tested the effects of these compounds on Sequenase, an engineered version of T7 DNA polymerase lacking 3′-to-5′ exonuclease activity. These compounds did inhibit Sequenase, albeit with IC_50_ values 50-fold greater than those observed for RT (data not shown). We also looked at transcription by T7 RNA polymerase and found no apparent inhibition by these compounds (data not shown). Hence we inferred, at the time, that these compounds targeted DNA polymerases, especially RT.

**Figure 3:**
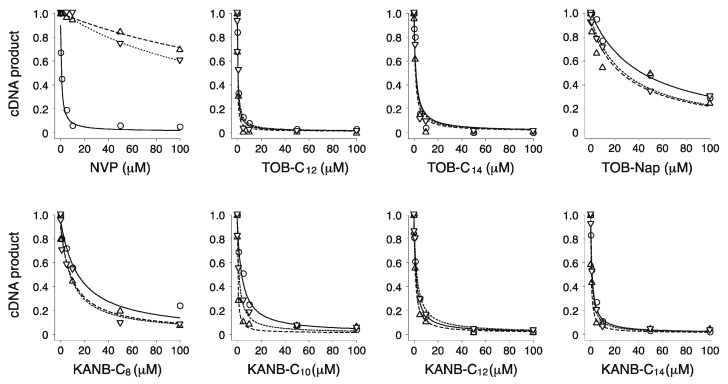
Modified aminoglycosides are potent inhibitors of wild-type (WT) and NNRTI-resistant HIV-1 RT. Effects of various compounds (as indicated) on the yield of cDNA product in primer extension reactions containing WT (◯), K103N (▽), or Y181C (△) RT enzymes. Data were fit to a modified dose-response equation to obtain the values shown in Table 1.

We purified wild-type (WT) and two mutant versions of HIV-1 RT and tested their susceptibility to inhibition by the TOB derivatives (Figure 3, Table 1). Substitution K103N and Y181C each confers resistance to multiple non-nucleoside reverse transcriptase inhibitors (NNRTIs) and are among the most frequently isolated NNRTI-resistant mutations [25]. As expected, nevirapine (NVP, a well-known NNRTI) strongly inhibited the WT enzyme (IC_50_ = 0.3 µM) and had a much smaller impact on the K103N (IC_50_ = 180 µM) or Y181C variants (IC_50_ = 200 µM). The TOB derivatives TOB-C_12_, TOB-C_14_, and TOB-Nap strongly inhibited HIV-1 RT, with IC_50_ values ranging from 0.3 to 40 µM (Table 1). Inhibition by these compounds was virtually unaffected by K103N or Y181C, suggesting a mechanism distinct from that of NNRTIs.

We made analogous derivatives of kanamycin B (KANB), an AG structurally similar to TOB, and tested their activities against HIV-1 RT. There is a single chemical difference between KANB and TOB in ring I: KANB has a 3′-hydroxyl group (R_1_ = OH), whereas TOB is devoid of a substituent at this position (R_1_ = H) (Figure 1). Four derivatives of KANB were analyzed, with 6”-linear alkyl substituents (C_8_, C_10_, C_12_, and C_14_). All four of these compounds inhibited HIV-1 RT, with IC_50_ values ranging from 0.5 to 8.0 µM (Figure 3 and Table 1). Neither K103N nor Y181C conferred resistance to these compounds, as indicated by equivalent or slightly smaller IC_50_ values. These data are highly similar to those obtained for the TOB derivatives, indicating that the chemical difference in ring I has essentially no influence on inhibitory activity.

**Figure 4:**
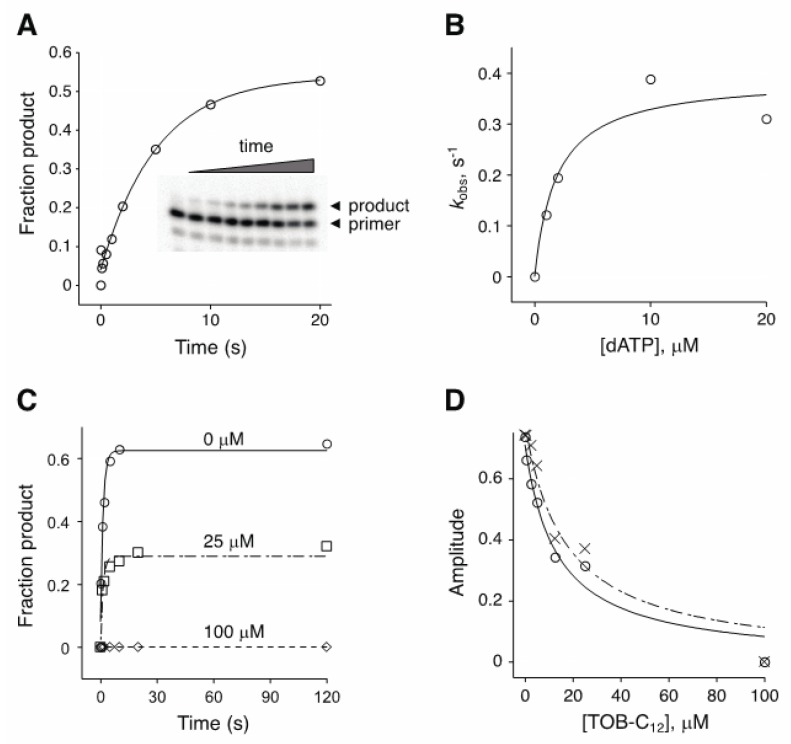
Effect of TOB-C_12_ on the kinetics of single-nucleotide incorporation by AMV RT. (**A**) Example of an experiment measuring the rate of adenosine monophosphate incorporation by AMV RT upon addition of dATP (2 μM). Primary data (inset) were quantified, plotted versus time, and fit to a single-exponential function to obtain the observed rate (*k*_obs_). (**B**) A secondary plot of *k*_obs_ versus dATP concentration suggests a *K*_M_ (dATP) of ~2 μM. (**C**) Primer:template was incubated with or without TOB-C_12_ prior to rapid mixing with dATP (150 μM) and quenching at various times. Time courses of product formation in the absence (◯) or presence (☐, 25 μM; ◇, 100 μM) of TOB-C_12_ are shown. (**D**) Amplitude was measured as a function of TOB-C_12_ concentration, in reactions containing 10 μM (◯) or 150 μM (✕) dATP.

The experiments above involved multiple-nucleotide extension of a primer annealed to an RNA (or DNA) template. To further investigate the basis of inhibition, we analyzed the kinetics of single-nucleotide incorporation (Figure 4). In the absence of inhibitor, the observed rate (*k*_obs_) of adenosine monophosphate addition by AMV RT increased with dATP concentration, yielding a *K*_M(dATP)_ value of ~2 µM (Figure 4B). Inclusion of TOB-C_12_ with RT and primer:template prior to rapid mixing with dATP reduced the amplitude of the reaction without affecting the rate (Figure 4C). The amplitude of the reaction decreased as function of TOB-C_12_ concentration, yielding under these conditions IC_50_ values of 13 µM and 17 µM for reactions containing dATP at 10 µM or 150 µM, respectively (Figure 4D). The fact that only amplitude was reduced suggests that binding of TOB-C_12_ completely blocks nucleotide incorporation in some way.

### 2.2. AG variants Bind Nucleic Acids in High-Molecular-Weight Complexes

**Figure 5:**
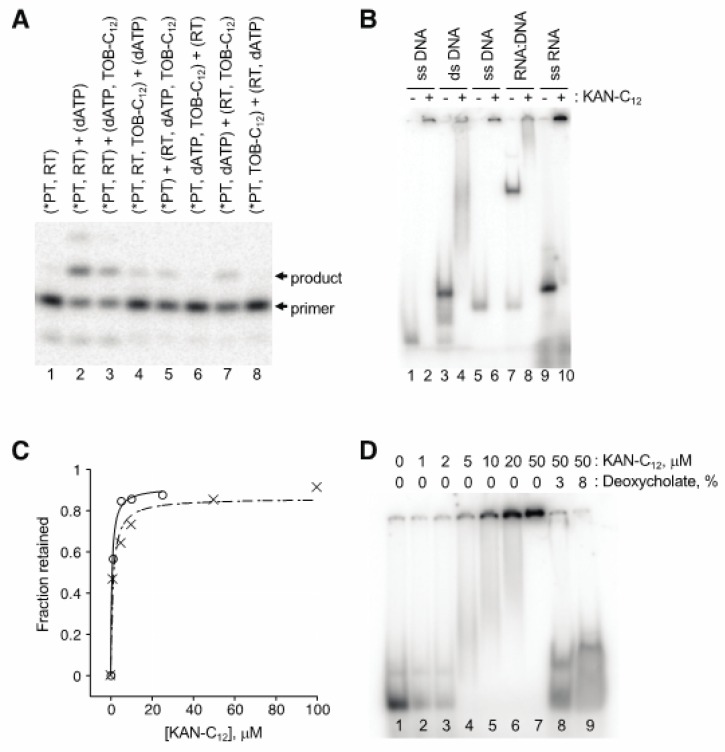
Modified aminoglycosides bind nucleic acids in high-molecular-weight complexes. (**A**) Preincubation of TOB-C_12_ with primer:template duplex results in the largest degree of inhibition. Components of the single-nucleotide primer-extension reaction (radiolabeled primer annealed to RNA template, *PT; HIV-1 reverse transcriptase, RT; dATP; TOB-C_12_) were pre-equilibrated in separate tubes (denoted by parentheses) for 20 min at 37 °C and then combined (as indicated) and further incubated for 1 min prior to PAGE analysis. (**B**) Gel-shift experiments show that KAN-C_12_ forms high-molecular weight complexes with various nucleic acids. A radiolabeled DNA oligonucleotide (18mer, lanes 1–4; 22mer, lanes 5–8), alone or pre-annealed to a complementary strand (as indicated), or an RNA oligonucleotide (25mer, lanes 9–10), was incubated with or without KAN-C_12_ (150 μM) for 15 min at 25 °C (as indicated) and then subjected to native PAGE. (**C**) Experiments with ultrafiltration devices show that RNA or DNA oligonucleotides form large and stable complexes. Radiolabeled oligonucleotide (DNA, ◯; RNA, ✕) was incubated with KAN-C_12_ (various concentrations) and the fraction in complexes of >300 kDa was determined by ultrafiltration (300 kDa MWCO). Data were plotted and fit to the equation y = (y_max_∙x)/(*K*_D_ + x) to yield apparent *K*_D_ values (0.6 μM, 1.1 μM). (**D**) Ionic detergents disrupt complexes. A radiolabeled RNA oligonucleotide was incubated with KAN-C_12_ (various concentrations, as indicated), and complexes formed at 50 μM KAN-C_12_ were subsequently incubated with deoxycholate (as indicated) prior to native PAGE analysis.

AGs are well known to bind RNA, raising the possibility that these compounds act by targeting the template or primer:template rather than RT. Our first clue that this was the case came from observation that nucleic acid “competitor” substantially reduced the degree of inhibition. A four-fold excess of competitor oligonucleotide (2 µM) over primer-annealed template (0.5 µM) increased the IC_50_ from 2.7 µM to 5.7 µM in a conventional primer extension assay of AMV RT (data not shown). Order-of-addition experiments provided further evidence that these AG compounds target the primer:template. An example of one such experiment, involving single-nucleotide incorporation by HIV-1 RT, is shown in Figure 5A. When TOB-C_12_ was preincubated with primer:template in the absence of RT, no detectable product was seen upon combining the remaining components (lanes 6 and 8). On the other hand, preincubation of TOB-C_12_ with RT hindered product formation to a lesser degree (lanes 5 and 7).

Gel mobility shift experiments demonstrated that these AG variants bind nucleic acids, forming high-molecular-weight complexes (Figure 5B). When a radiolabeled 18mer DNA oligonucleotide was preincubated with KANB-C_12_ (100 µM) and subjected to native gel electrophoresis, all signal was shifted to the well, with no band corresponding to free oligonucleotide (compare lanes 1 and 2). A DNA duplex, formed by annealing the 18mer to a 31mer, migrated more slowly through the gel (lane 3). Preincubation with KANB-C_12_ resulted in loss of this band, with signal shifted higher in the gel and/or in the well (lane 4). Similar results were seen when other oligonucleotides were tested, including an RNA oligonucleotide (Figure 5B, lanes 5–10).

Next, we used ultrafiltration devices to further evaluate the stability and nature of these complexes. Radiolabeled DNA or RNA oligonucleotide was incubated with KANB-C_12_ (various concentrations), and the amount of nucleic acid able to pass through the porous membrane was quantified. The fraction of retained (bound) oligonucleotide increased sharply as a function of KANB-C_12_, yielding binding curves with *K*_D_ values of ~ 1 µM (Figure 5C), reminiscent of the IC_50_ values reported above for the same compound (Table 1). Devices with molecular weight cut off (MWCO) of 30 kDa or 300 kDa gave virtually identical results, indicating large complexes, consistent with the gel mobility shift data above. Analogous experiments performed with duplex DNA or heteroduplex DNA:RNA yielded similar data with no indication of enhanced complex stability (data not shown).

The AG variants analyzed here are amphiphilic, raising the possibility that they form micelles and/or bilayer vesicles in aqueous solution, which bind nucleic acids on their surfaces. In line with this hypothesis, ionic detergents such as SDS and deoxycholate readily disrupted these complexes (Figure 5D), whereas high concentrations of urea, guanidine, KCl, or EDTA had little or no impact.

## 3. Discussion

Here, we report the serendipitous finding that AG variants bearing C_12_ or C_14_ linear alkyl substituents are potent inhibitors of RT. These compounds act by binding nucleic acids with high affinity, forming high-molecular weight complexes. Given the amphiphilic nature of these AG derivatives, we suspect that they form micelles and/or vesicles with surface-bound nucleic acids. In line with this view, the complexes dissociate when challenged with ionic detergents.

Natural AGs have highest affinity for the narrow major groove of A-form RNA and exhibit progressively lower affinity for nucleic acid duplexes as the major groove width increases [26]. Our modified AGs bind similarly to DNA or RNA, in single- or double-stranded form, implying a helix-independent mode of binding. In either a micelle or vesicle, the hydrophilic surface would present an array of AG moieties. We envision that multiple interactions between the positively-charged amino groups on this surface and the negatively-charged phosphates on the nucleic acid backbone results in the tight and nonspecific overall binding that we observe.

Early on in this work, we mistakenly deduced that these compounds targeted RT, because other polymerases appeared less susceptible or resistant to these compounds. Why potent inhibition was only seen in the RT assays remains unclear. This might be due to differences in experimental conditions or distinct abilities of the polymerases to compete for primer: template. In any event, it is clear that these AG variants bind nucleic acids and thereby inhibit RT.

Our findings may hold particular relevance in the realms of biotechnology and chemical engineering. These AG derivatives could be useful for localizing nucleic acids to surfaces or delivering nucleic acids to cells or cellular compartments. Indeed, independent studies by Pitard, Lehn, and coworkers have shown that analogous molecules containing an AG head group linked either to cholesterol or dual aliphatic chains (e.g., C_18_) can be used to deliver nucleic acids into cells [27,28,29]. Such designer molecules form various high-order structures with DNA or RNA in vitro, which efficiently transfect diverse cell lines with plasmid DNA, mRNA, or siRNA. A deeper understanding of the unique chemical and biophysical properties of these compounds may lead to new applications or improved technologies down the road.

## 4. Materials and Methods

### 4.1. Biochemical Reagents

Plasmids pET-RT66 and p6H-RT51, which overexpress HIV-1 RT subunits p66 and His_6_-tagged p51, were generously provided by Samuel Wilson (NIH). Mutations K103N and Y181C were each introduced into pET-RT66 using QuikChange^TM^ mutagenesis. Subunits p66 and His_6_-p51 were overproduced in *E. coli* strains BL21/DE3 and DH5α, respectively, and the RT heterodimer (WT or mutant forms) was reconstituted in mixed lysates and purified essentially as described [30]. Each overexpression strain was grown in LB medium with appropriate antibiotics at 37 °C until the culture reached an OD_600_ of 0.6, IPTG (0.5 mM) was added, and the culture was further incubated for 3.5 h to allow ample protein production. Cells (from 4 L culture) were pelleted and stored at −20 °C. All subsequent steps were performed at 4 °C. Each cell pellet was resuspended in 20 mL buffer A [50 mM Tris-HCl (pH 7.9), 60 mM NaCl, 10% glycerol, 1 mM βME, and protease inhibitors (Protease Inhibitor Cocktail, Roche Diagnostics)], and the cells were lysed using a French Press. The lysate was clarified by centrifugation at 5900 g for 15 min, and the protein concentration of each lysate was estimated using the Bradford assay. Based on these estimates, each p66 strain lysate was separately mixed with p51 strain lysate in a 3:1 ratio. Each mixture was passed through a Q-Sepharose column (2 mL, Bio-Rad), pre-equilibrated with buffer A, and the flow-through (containing HIV-1 RT) was collected. NaCl (0.5 M), imidazole (10 mM), and 4 mL of His-Bind nickel column resin (Ni-NTA Agarose, QIAGEN) pre-equilibrated in buffer B [50 mM Tris-HCl (pH 7.9), 500 mM NaCl, 10 mM imidazole, 10% glycerol, and 1 mM βME] were added, and the mixture was rotated for 1 h. The resin was poured into a column and washed with 10 column volumes (CV) of buffer B, 10 CV of buffer C [50 mM Tris-HCl (pH 7.9), 1 M NaCl, 10 mM imidazole, 10% glycerol, and 1 mM βME], and 10 CV of buffer D [50 mM Tris-HCl (pH 7.9), 1 M NaCl, 15 mM imidazole, 10% glycerol, and 1 mM βME]. Proteins were eluted from the column with an imidazole gradient (10–500 mM) in buffer B. Fractions (0.5 mL) were collected, and DTT (2 mM) and EDTA (5 mM) were added to each fraction. Fractions were analyzed by SDS-PAGE, and those containing p66 and p51 at a 1:1 ratio were pooled. The RT was further purified using a FPLC RESOURCE S column (6 mL, GE Healthcare). The protein was bound to the column, washed with 10 CV of buffer E [50 mM Tris-HCl (pH 6.5), 60 mM NaCl, 10% glycerol, and 1 mM βME], and eluted using a NaCl gradient. Fractions (0.5 mL) containing purified RT with p66 and p51 subunits at a 1:1 ratio were pooled, dialyzed against buffer F [50 mM Tris-HCl (pH 7.5), 100 mM NaCl, 1 mM βME, and 10% glycerol], and stored at −20 °C.

Template RNA, based on T4 *gene 32* [m291 or m292, [31]; most experiments] was made by in vitro transcription and PAGE purified. Primer (22 nt) complementary to the 3′-end of template RNA was 5′-end labeled using T4 polynucleotide kinase (NEB) and γ-[^32^P]-ATP. AMV RT was purchased from Life Sciences Advanced Technologies, Inc. Sequenase was purchased from Affymetrix/Thermo Fisher Scientific. NVP (obtained from the NIH) was dissolved in DMSO. AG derivatives, synthesized as described previously [20,32], were dissolved in water.

### 4.2. Primer Extension Assays

To assay multi-nucleotide incorporation by RT, 5′-[^32^P]-labeled primer (~0.05 µM) was annealed to mRNA (0.33 µM, unless otherwise indicated), RT (HIV-1 or AMV; 32 nM) and the four deoxynucleotide triphosphates (dNTPs; 375 µM each) in buffer G [10 mM Tris-HCl (pH 7.5), 60 mM NH_4_Cl, 10 mM MgCl_2_, and 6 mM βME] were added, in the absence or presence of inhibitor (as indicated), and reactions were incubated at 37 °C for 10 min. An equal volume of stop buffer (95% formamide, 20 mM EDTA, 0.05% xylene cyanol, and 0.05% bromophenol blue) was added, and products were analyzed by denaturing 7% PAGE. The relative amount of the full-length cDNA product (y) was plotted as a function of inhibitor concentration, and the data were fit to the modified dose response equation y = *a* + *b*/[1 + (x/*c*)] (with Hill coefficient of 1), where *a* corresponds to background signal, *b* is the maximal cDNA product observed, and *c* is the IC_50_ value.

Single-nucleotide incorporation by RT was measured using a quench-flow machine (KinTek RQF-3). Typically, 5′-[^32^P]-labeled primer (<0.05 µM) was annealed to mRNA (0.2 µM), equilibrated in buffer H [Tris-HCl (pH 7.5), 80 mM KCl, 20 mM MgCl_2_, 2 mM DTT] with RT (AMV or HIV-1, as indicated) in the absence or presence of inhibitor (as indicated), and rapidly mixed with dATP (variable concentration, as indicated). Each reaction was quenched with 0.5 M EDTA at various time points; the data were plotted and fit to a single exponential equation to obtain observed rate and amplitude.

To assay Sequenase activity, two DNA oligonucleotides (5′-GGAATTCACTAGTTTGAAATGAATGAAGCACTCTACTATATTCTTAATAGGTCC-3′ and 5′-CGGGATCCATTTCTCGAGGGATATGATAGTCAAACAGGACCTATTAAG-3′, 0.5 µM each) with complementary 3′-ends were annealed in Sequenase buffer [40 mM Tris-HCl (pH 7.5), 20 mM MgCl_2_, 50 mM NaCl]. DTT (1 mM) and dNTPs (375 µM each) were added, followed by inhibitor (as indicated, various concentrations) and Sequenase 2.0 (30 nM; USB). Reactions were incubated at 37 °C for 10 min, and DNA products were resolved by 7% denaturing PAGE and quantified. Data were analyzed as described above for the RT multi-nucleotide incorporation assay.

### 4.3. Binding Experiments

For binding reactions, components were incubated in buffer H at 25 °C for 15 min. The fraction of bound 5′-[^32^P]-labeled nucleic acid was evaluated using native 6% PAGE or ultrafiltration devices (Nanosep Omega, PALL Life Sciences).

## 5. Conclusions

In this work, we show that AG derivatives carrying C_12_ or C_14_ alkyl substituents are potent inhibitors of RT. These compounds act by binding nucleic acids in high-molecular-weight complexes. As these compounds are amphiphilic, they likely form micelles and/or vesicles with surface-arrayed AG moieties that interact favorably with the sugar-phosphate backbone of nucleic acid strands.

## Figures and Tables

**Table 1:** Inhibition of WT and mutant HIV-1 RT by various compounds.

Compounds	IC_50_ (µM)		
	WT	K103N	Y181C
NVP	0.3 ± 0.2	180 ± 40	200 ± 30
TOB-C_12_	0.3 ± 0.1	0.6 ± 0.1	0.5 ± 0.1
TOB-C_14_	1.9 ± 0.3	1.2 ± 0.6	1.4 ± 0.4
TOB-Nap	40 ± 5	40 ± 11	12 ± 4
KANB-C_8_	8 ± 3	7 ± 1	8 ± 2
KANB-C_10_	3.8 ± 0.3	1.9 ± 0.3	1.0 ± 0.6
KANB-C_12_	1.9 ± 0.3	2.0 ± 0.2	0.5 ± 0.3
KANB-C_14_	1.0 ± 0.1	0.9 ± 0.1	0.7 ± 0.3

Data represent the mean ± SEM from 3 or more independent experiments.
